# HIV Due to Female Sex Work: Regional and Global Estimates

**DOI:** 10.1371/journal.pone.0063476

**Published:** 2013-05-23

**Authors:** Annette Prüss-Ustün, Jennyfer Wolf, Tim Driscoll, Louisa Degenhardt, Maria Neira, Jesus Maria Garcia Calleja

**Affiliations:** 1 Department of Public Health and Environment, World Health Organization, Geneva, Switzerland; 2 Sydney School of Public Health, Sydney Medical School, The University of Sydney, Sydney, Australia; 3 National Drug and Alcohol Research Centre, University of New South Wales, Sydney, Australia; 4 Centre for Health Policy, Programs and Economics, School of Population Health, University of Melbourne, Melbourne, Australia; 5 Department of HIV/AIDS, World Health Organization, Geneva, Switzerland; Public Health Agency of Barcelona, Spain

## Abstract

**Introduction:**

Female sex workers (FSWs) are at high risk of HIV infection. Our objective was to determine the proportion of HIV prevalence in the general female adult population that is attributable to the occupational exposure of female sex work, due to unprotected sexual intercourse.

**Methods:**

Population attributable fractions of HIV prevalence due to female sex work were estimated for 2011. A systematic search was conducted to retrieve required input data from available sources. Data gaps of HIV prevalence in FSWs for 2011 were filled using multilevel modeling and multivariate linear regression. The fraction of HIV attributable to female sex work was estimated as the excess HIV burden in FSWs deducting the HIV burden in FSWs due to injecting drug use.

**Results:**

An estimated fifteen percent of HIV in the general female adult population is attributable to (unsafe) female sex work. The region with the highest attributable fraction is Sub Saharan Africa, but the burden is also substantial for the Caribbean, Latin America and South and Southeast Asia. We estimate 106,000 deaths from HIV are a result of female sex work globally, 98,000 of which occur in Sub-Saharan Africa. If HIV prevalence in other population groups originating from sexual contact with FSWs had been considered, the overall attributable burden would probably be much larger.

**Discussion:**

Female sex work is an important contributor to HIV transmission and the global HIV burden. Effective HIV prevention measures exist and have been successfully targeted at key populations in many settings. These must be scaled up.

**Conclusion:**

FSWs suffer from high HIV burden and are a crucial core population for HIV transmission. Surveillance, prevention and treatment of HIV in FSWs should benefit both this often neglected vulnerable group and the general population.

## Introduction

Female sex workers (FSWs) suffer from relatively high risk of infection with the human immunodeficiency virus (HIV) [1]. Sex work, defined here as “the provision of sexual services in exchange for money, goods, or other benefits” [2], is structured differently around the world, and comprehensively capturing this heterogeneous group of workers may be difficult. This is particularly the case when they are stigmatized or even illegal, which might make FSWs reluctant to disclose their profession. The definition also includes many women who sell sex occasionally or for benefits other than money (often called ‘indirect’ FSWs as opposed to ‘professional’, ‘brothel-based’ or ‘direct’ FSWs) and might not consider themselves as sex workers. These women are unlikely to appear in population statistics [2]. FSWs may have an important role in disease transmission in a population, often spreading the virus between other key population groups or clients who subsequently transmit the disease to their spouses [3]. UNAIDS has recently prioritized HIV prevention focusing on key populations [4]. A recent systematic review and meta-analysis on low and middle income countries found an overall HIV prevalence in FSWs of 12% and an odds ratio for HIV infection of 13.5 compared to other women of reproductive age [5]. However, the fraction of HIV infections in the general female population that is attributable to the occupational exposure of female sex work, i.e. adjusted for other risks in FSWs, (as opposed to total HIV in sex workers), is currently unknown.

In general, the two main risk factors for HIV transmission in a population are unprotected sex and injecting drugs with contaminated instruments [6]. Globally, most HIV infections are transmitted through heterosexual transmission [7]. However, in certain regions including Eastern Europe and Central Asia the leading cause for infection is injecting drugs [4]. FSWs are frequently also people who inject drugs (PWID) which makes them particularly more vulnerable to HIV transmission [8]–[10]. Prevention of HIV infection in FSWs not only protects their health but also prevents onward transmission to clients and their partners.

The objective of this work was to determine the proportion of HIV prevalence in the general female population that is attributable to occupational exposure in FSWs, and thereby (1) estimate the public health importance of this risk factor and (2) highlight the potential impact of preventive action targeted towards this occupational group. Male sex workers are also an important at-risk population, however available data on this group were too scarce to include them in our analysis.

## Methods

### Data Sources

Data on HIV prevalence in women aged 15 or older in the general population (from now on called the ‘general female adult population’) were taken from the UNAIDS Global Reports [11], [12]. Data on HIV in special key populations (e.g. FSWs, PWID) were also taken from the UNAIDS Global Reports [11], [12] and were completed with all Country Progress Reports related to the United Nations Special Session (UNGASS) available on http://www.unaids.org/en/regionscountries/countries/ [13] and are listed in Table S2. Further information on the primary sources and methods underlying these prevalence data can be found on the UNAIDS web site (www.unaids.org/en/dataanalysis/). Data from individual studies or surveys, focusing on non-representative areas within a country, were not included. We extracted data covering the period from 2000 to 2012 as a basis for developing a time series. The population numbers of FSWs and PWID were extracted from major global reviews [2], [14], [15]. A further search in Medline and Google over the last ten years identified country and regional information on HIV prevalence in female PWID and FSWs injecting drugs, proportions of PWID selling sex, the female proportion of PWID and the proportion of HIV prevalence in non-injecting FSWs [14]–[23]. We used the following search terms (both as Mesh and keyword terms) “sex workers”, “sex work”, “prostitutes”, “prostitution”, “intravenous substance abuse”, “drug use” and “HIV infections”. The reference sections of such identified publications were screened for further sources.

Data on the overlap between sex work and injecting drug use were scarce and not available for a number of countries. We extrapolated the average regional value to these countries. Regions without suitable data were North Africa and Middle East, North America, the Caribbean and Australasia. For these regions, the mid-point of the global estimate of 42% PWID engaging in sex work was used [24]. Eligible studies are presented in Table S1.

### Calculation of the Population Attributable Fraction of HIV in the General Female Adult Population due to Female Sex Work

The population attributable fraction (PAF) is the proportion of disease in a population that is attributable to exposure to a risk factor. The PAF indicates the amount of disease in the population that could be prevented if exposure to the risk factor was eliminated [25], [26].

Our aim was to estimate the relative importance of female sex work for the general female adult population for the year 2011, while adjusting for an estimated proportion of HIV in FSWs due to injecting drugs. The proposed methods are based on the difference in HIV prevalence between female sex workers and the general female population, reduced by the HIV prevalence estimated to be due to IDU (for details see Texts S2 and S3). Intravenous drug use (IDU) is usually more frequent in sex workers than in the general population [24], [27]–[30] and can therefore confound and overestimate the association between sex work and HIV infection. The risk of HIV infection from a contaminated needle or syringe is much higher (around 1 in 125 unsafe injections) than the risk from a heterosexual act with an infected partner (around 1 in 2000 to 5000 acts) [31]. In light of limited data availability, especially on the overlap between sex work, drug use and HIV infection, we did not stratify FSWs by IDU but assumed that all HIV in drug-injecting FSWs was due to IDU, and deducted this proportion of HIV infections from the final PAFs. We further assumed that, after excluding this proportion, all excess HIV prevalence in FSWs was attributable to sex work.

### Modelling of HIV Prevalence in FSWs for the Year 2011

Data for HIV prevalence in FSWs are needed for the calculation of the PAFs. However, these data are not available for all countries, and, if available, cover different years and often do not include 2011. The number of data points by country ranged from zero to eight. In total, we identified 214 data points for HIV prevalence in FSWs for the period between 2000 and 2012.

To estimate HIV prevalence in FSWs for countries for the year 2011, we used different methods depending upon whether country-level HIV data were available for FSW. For countries with at least one data point for HIV prevalence in FSWs we used a linear two-level model [32] with a random intercept by country and a covariate for region (WHO 14 sub-regions [33]) to estimate HIV prevalence in FSWs in the year 2011 (see details in Text S4).

For countries with no data on HIV prevalence in FSWs (n = 20), we estimated HIV prevalence in FSWs in the year 2011 using a linear model regressing HIV prevalence in FSWs in 2011 on HIV prevalence in the general, female, adult population in the year 2011. The regression was stratified by region. Prior to this, high correlation (Pearson’s product-moment correlation coefficient 0.8, p<0.0001) between HIV prevalence in the general female adult population for 2011 and the estimated HIV prevalence in FSWs for 2011 was observed. The correlation was consistent among regions.

### Uncertainty

The PAF is sensitive to uncertainties in the parameters used for its calculation [34]. Sensitivity analyses were performed to explore the impact of likely variations in the individual estimates. Varied estimates were (a) the upper and lower estimates of HIV prevalence in the female adult population by country [35], (b) the upper and lower estimates of the proportion of PWID who sold sex, (c) a 20% variation in the proportion of HIV in FSWs, as has been done previously [36]. Confidence intervals were also calculated using Markov Chain Monte-Carlo (MCMC) sampling. However, the latter confidence intervals were consistently smaller than the variations obtained with sensitivity analysis. Given the uncertainty in much of our data, we decided to present results from the sensitivity analysis, thereby maximizing estimated uncertainty. Uncertainty estimates obtained with MCMC can be requested from the corresponding author.

## Results

We estimated PAFs for female sex work as a risk factor for HIV in women for 137 countries, covering around 99% of the world population in 2011. The estimated HIV prevalence in the general female adult population attributable to female sex work (in FSWs), and the upper and lower estimates obtained from the sensitivity analysis, are summarized in Table 1. Globally, 15% of HIV infections are attributable to the occupational risk factor female sex work. HIV from female sex work is most important in Sub Saharan Africa where 18% of HIV prevalence is attributable to this risk factor. The PAFs are also high in the Caribbean, Latin America and South and Southeast Asia (between 7 and 9%). In East Asia, and North Africa and the Middle East, the fraction of HIV attributable to female sex work amounts to around 3%, while it lies below 2% in Europe, North America and Oceania. High-income regions display the lowest contribution of female sex work to the HIV epidemic.

**Table 1:** Proportion of HIV and number of deaths in women above 15 years attributable to sex work.

	Proportion of HIV in the general female population (aged15+) that is attributable to sex work in FSWs (%)		Number of deaths attributable to female sex work (2011)
Region	PAF [%]	upper/lower bounds from sensitivity analysis [%]	Deaths [n]
Sub-Saharan Africa	17.8	13.6–22.1	98,100
Latin America	7.0	3.9–9.9	1,100
North America	0.6	0.4–0.8	<100
Asia, East	3.5	2.2–5.4	500
Asia, South and Southeast	7.4	5.7–9.2	5,900
Caribbean	9.0	7.6–11.0	400
Europe East and Asia, Central	0.8	0.7–1.0	200
Europe West and Central	1.6	1.4–2.2	<100
North Africa, Middle East	3.1	2.3–3.8	200
Oceania	0.1	0.1–0.1	<100
**Global**	15.0	11.5–18.6	106,400

PAF: Population attributable fraction.

If the estimated PAFs are applied to the latest HIV death estimates on HIV/AIDS for the year 2011 ([37]: unpublished estimates), the number of deaths attributable to female sex work would amount to 106,000 globally in 2008, with an overwhelming 98,000 in Sub-Saharan Africa. Another 5,900 deaths are estimated to occur in South and South-East Asia and 1,100 in Latin America, while in the other regions the number of deaths from HIV in FSWs is expected to remain below 1,000 per year. The number of cases of HIV in FSWs in developed countries mostly seems to be negligible.

The input data on HIV prevalence by country in the general female population and in the key populations of sex workers and IDUs, as well as the size of those populations, are provided in the Webappendix.

## Discussion

### Discussion of Results

Fifteen percent of the global HIV burden in women is attributable to the occupational risk factor female sex work, resulting in more than 100,000 deaths per year. The regions with the highest PAFs are Sub Saharan Africa, the Caribbean, and South and Southeast Asia. Previous research confirms a high disease burden of HIV infections in FSWs. In fifty low and middle income countries overall HIV prevalence was 12% and 31% in 26 countries with medium and high background HIV prevalence [5]. Our analysis contributes to the evidence in presenting the proportion of HIV in the female population that occurs in FSWs and that is directly attributable to occupational exposure to sex work, and is updated with the latest evidence available for the year 2011.

Our estimates refer only to the proportion of HIV infections that is due to sex work in FSWs. They do not describe how much disease is caused in people other than FSWs that originates from female sex work, i.e. infection of clients, spouses of clients, partners and finally their children through maternal transmission. The fraction of HIV infections in the general (male and female) population due to female sex work in all population groups would therefore be much higher. A multicountry analysis in West Africa concluded that 15% to 25% of new HIV infections are infections in FSWs and their clients [36]. Another 20% to 30% occurred in, often low-risk, partners of people with high risk behavior. In Accra, the capital of Ghana in West Africa, 84% of HIV prevalence in the male population aged 15 to 59 years was attributed to sexual contact with sex workers [38]. In Cotonou, the capital of Benin, it was postulated that nearly all HIV infections in women and 76% in men were due to sexual contacts with FSWs [34], [39]. The rapid and early spread of HIV in some Asian countries like Thailand and Cambodia has been associated with a high use of commercial sex [40]. A recent Indian nationally representative survey found that about 4% of Indian men visited a FSW in the previous year with much higher percentages in regions with high HIV prevalence [41]. DHS surveys reporting the percentage of men who admitted to having had sex with a sex worker in the last year found a wide range of values between approximately 0.1 to over 10% [42]. Proportions from surveys will often underestimate the true value as risky behavior has been shown to be considerably underreported [36].

Particularly in developing countries, HIV prevalences in FSW were relatively high. The region with the highest burden both in FSWs and the general population is Sub Saharan Africa. This was also seen in previous research [2]. FSWs are important contributors to the transmission of HIV in a population. In a country, HIV epidemics often spread initially among key populations like PWID or sex workers. FSWs usually have high numbers of sexual partners and transmit the disease to other key populations (PWID, boyfriends, pimps) and to their clients [36]. Though FSWs have been shown to use condoms more often in commercial than in private sexual contacts [36], unprotected sex involving FSWs remains common in certain regions [43]. Clients of FSWs transmit the virus to their spouses or other sexual partners, acting as a bridging population between key populations and the general population [44]. In Benin, for example, clients of FSWs were frequently married and had other sexual partners, which implies a wide circle of potential subsequent infections [39]. A very low proportion of those men used a condom with both FSWs and other sexual partners. Sexual partners of clients, e.g. spouses with often a low-risk behavior, can therefore be at high risk of HIV infection [39]. The disease might finally be transmitted to children via vertical, maternal transmission [40]. In light of these dynamics, HIV prevalence in sex workers has been described as an “early warning system” for transmission to the general population where the disease is not yet or not any more generalized [44]. Nowadays, overall HIV incidence is decreasing in over 40 countries whereas it is stable in almost 20 countries or increasing in other key populations, but mostly in some specific regions [35], [45]. Countries with a high percentage of men using the services of FSWs and low condom use are at risk of (re)spreading infections from those concentrated epidemics to the general population.

Disease prevention can be targeted either at the general population or at key population groups. A systematic review and meta-analysis on the importance of sexual risk factors in different stages of the HIV epidemic (i.e. low-level, concentrated, generalized, for a definition of these stages see [44]) emphasized the general benefits in targeting FSWs as an important core group for HIV transmission [46]. Opposing previous opinion [44], female sex work was found to be as important in HIV transmission in generalized epidemics as in low-level or concentrated epidemics [46], which is also in line with mathematical HIV modeling [47]. Another possibility for prevention is to target clients of sex workers and it was postulated that focusing on both FSWs and clients would increase the effectiveness of prevention efforts [48].

Interventions to reduce HIV transmission in FSWs (and/or their clients) have so far included behavioral interventions (e.g. condom promotion/availability, empowerment of FSWs, health education) [49], prevention of mother-to-child transmission, circumcision of the male population and now increasingly also medical prevention through early antiretroviral therapy, preexposure chemoprophylaxis and antiretroviral microbicides [45]. Those interventions are especially important as no vaccine is available now or likely in the near future to prevent HIV infections [45].

For instance two countries Cambodia and Thailand, which had an early and rapid rise of the HIV epidemic, implemented highly successful prevention programmes that were associated with an increase in condom use in commercial sex to around 90% [40], [50]. The subsequent decline in HIV prevalence in the general population was largely attributed to these interventions. Similarly, in many African countries health education, condom promotion, STI screening and treatment were successfully implemented, increasing safer-sex practices and lowering HIV/STI transmission [51]–[54]. A systematic review of interventions with FSWs in Africa, Asia and Latin America found that condom promotion, health education and improved STI management effectively prevented HIV and STIs [55]. A Cochrane systematic review on behavioral interventions in FSWs in low- and middle-income countries concluded that those interventions effectively prevented HIV and STIs, though evidence was limited [49]. For high-income countries no evidence on the effectiveness of behavioral interventions on HIV incidence or prevalence was found [56].

We only estimated the proportion of the HIV burden due to female not male sex work in this analysis. Male sex workers are an important at-risk population and often have high HIV prevalence rates [57]–[60]. However, available data on male sex workers were too scarce to include them in this analysis.

### Discussion of the PAF as Opposed to the Preventable Fraction

The PAF, as calculated for this paper, of a certain risk factor is the amount of disease in the general population that could be avoided if exposure to this risk factor was removed. This fraction can therefore be interpreted as the maximum, theoretically achievable disease reduction in terms of this risk factor. In our example, HIV prevalence in the general female adult population would shift to the prevalence of a population with no (unsafe) female sex work. However, it may not be possible to completely prevent exposure or to make female sex work completely safe. Nevertheless, effective interventions against sexual transmission of HIV targeted at FSWs exist and have been successfully implemented in many countries. The PAF as calculated here cannot exactly be equated with the proportion of disease that can be prevented in real life. Certain interventions, however, have been shown to be highly effective [45], [50], and the low prevalence of HIV infections in FSWs in many high income countries shows that very low excess risk of transmission is possible in FSWs. The PAF as calculated here is therefore probably not far from the preventable fraction, and remains a useful measure to describe the relative importance of a risk factor in a population.

### Limitations

Limitations in our estimates need to be acknowledged. The main uncertainties in our results relate to (1) limited data availability on HIV prevalence in FSWs and PWID and on the overlap between sex work and IDU, great variation in definitions, assessment and sampling methods used, and limited country representativity of available data; (2) the use of data covering only part of the female sex worker population; (3) our conservative attribution of all HIV in FSWs practicing IDU to their injecting drug use; (4) other risk factors for HIV transmission; (5) the migration of FSWs between countries, with sex workers who were HIV positive in their country of origin working as sex worker in their country of emigration; (6) the turnover and population change of FSWs; (7) the failure to capture also secondary infections, e.g. from infected sex workers to clients and onwards, and (8) the assumption used that infected FSWs are equally likely to die from the infection as the general population.

First, the different definitions and data collection methods used, covering in varying extents the different FSW subgroups, increase uncertainty. The estimated population size of FSWs can differ considerably when the focus is only on ‘official’ versus ‘occasional’ FSWs, or on those based in specialized establishments versus ‘street-based’ FSWs, or on women selling sex for money versus for other goods are considered [2]. Sex workers are not a homogenous group and a previous literature search identified more than 20 different types [61]. Surveys or other assessments may capture primarily FSWs working in registered establishments, as compared to for example street-based sex workers [2], [62] who may be at higher risk of HIV transmission and also exposed to additional risks [63]. This would lead to an underestimation of the HIV burden attributable to female sex work. Furthermore, many assessments relating to FSWs take place in urban settings and may not be representative for the country due to within-country heterogeneity.

Second, much data collection on FSWs and PWID is done in larger urban centers or high-prevalence regions and may not be nationally representative, e.g. many studies in China have been conducted in Yunnan which is the most HIV affected region in China [64]–[67]. It is difficult to perform representative surveys in FSWs, as prostitution is often clandestine and FSWs therefore generally difficult to access [39], [44]. The absence of most of the data required for USA and Canada resulted in reliance on modeled data alone. Estimates for North America therefore are only indicative and need to be interpreted with special caution.

Third, an important risk factor for HIV infection in FSWs other than the sex work itself is IDU. As the infectivity of a shared contaminated needle is generally much higher than of unsafe sex with a HIV-positive partner, we assumed that in case of IDU in an HIV-positive FSW, infection was due to drug use. Besides being a confounder, IDU can however also be on the causal pathway between sex work and HIV [68]. Substance abuse has accordingly been considered elsewhere an occupational hazard in sex work [10], [69]. In FSWs where IDU would be considered a consequence of sex work, HIV prevalence would therefore ultimately be attributable to sex work. As we did not have information on the temporal sequence of sex work and intravenous drug use we deducted all HIV infections in FSWs that were also injecting drugs. Despite this uncertainty, this means our estimates of HIV attributable to sex work are again conservative.

Fourth, FSWs might be exposed to other risk factors than sex work and IDU that pose them at elevated risk of HIV infection, for example unprotected sex with partners [38], [39], [62] from high-risk groups and engage in more unsafe sex in private relationships than the general female adult population. Condom use in sexual acts involving FSWs is generally less common with boyfriends than with clients with elevated HIV prevalence [39]. However, engagement in sex work might in many cases have preceded contacts with high-risk sexual partners [34], [40], [44].

Fifth, another potential confounder is migration. FSWs may consist of a disproportionally high share of immigrated women [39], [70] who originated from countries with a higher HIV prevalence and who might have contracted the virus prior to engaging in sex work. Unfortunately, data to allow adjustment for migration were not available. The impact of immigrant sex workers on the estimates of HIV attributable to sex work is likely to be larger in developed countries, where the HIV prevalence is more likely to be lower than in the countries of origin of FSW. Still, FSW having a much higher risk of HIV infection than the general population [5], the effect of this potential confounder is probably limited. An example from Kenya has not shown any difference between the HIV rates in immigrated versus local FSWs [71]. In Western Europe, migrants constitute almost half of FSWs [72], however the number of HIV cases in FSW in that region is relatively negligible.

Sixth, the high turnover of FSW may introduce additional uncertainties [2]. High turnover may for example decrease the assessed HIV rate in FSWs as newcomers with background HIV rates may replace the FSW with higher HIV rates who drop out. Those FSWs who have contracted HIV during their occupation may furthermore increase the HIV rate in the general female population which they join as they drop out of their occupation. The latter effect may lead to underestimating the fraction of HIV due to sex work. The average duration of sex work may vary across regions, and is often limited to a few years. [73]. In addition, they move within countries, for example from rural to urban settings, or follow seasonal workers or tourists [2].

Seventh, the effect of transmission of HIV from sex workers to clients, spouses of clients, partners and their children through maternal transmission could not be taken into account due to limited data. Surveys performed in Sub-Saharan countries have shown that HIV infection from contact with sex workers can account for more than three quarters of all HIV infections in men in certain age groups in that region [36], [38]. This may be the largest limitation if we were to consider the full consequences of HIV infection from sex work.

Finally and eighthly, in applying the population attributable fraction to the total adult female HIV burden in order to estimate the burden attributable to sex work, it was assumed that FSWs with HIV have the same death rate from HIV as the general population. This may not be the case, however it was not possible to make any adjustments due to lacking data. This is not an uncommon assumption in burden of disease estimates due to often comparable data situations.

### Conclusions

Female sex work is an important risk factor for HIV infection. Fifteen percent of current HIV burden in the general female adult population is attributable to sex work. The great majority of deaths related to these infections are expected to occur in Sub-Saharan Africa, and also in South and South-East Asia. If we had considered all cases where infection originates from FSWs to other population groups (e.g. clients and their partners), the estimated attributable burden would be substantially elevated. Prevention programmes targeting FSWs have a great potential to lower disease burden in this key population and additionally decrease disease transmission in the general population.

HIV prevention is feasible and effective on a large scale [45]. Prevention remains most important in HIV control since no vaccine is available and access to treatment remains limited in many parts of the world. Consistent (male or female) condom use is the most effective disease prevention measure [45], [74] and would additionally reduce STI transmission which in turn would lower the risk for HIV transmission. Impressive HIV control has been achieved through nationally implemented condom promotion strategies [44], [50] and intervention strategies targeted at FSWs are cost-effective [75]–[77]. Prevention measures are enhanced when multiple prevention interventions are combined, e.g. condom use/availability, male circumcision and health education [45].

Current surveillance, medical care and prevention strategies for key population groups are insufficient [45]. HIV surveillance is crucial to monitor HIV and its dynamics as well as aiding priority setting and planning, implementing and conducting of prevention strategies [2]. FSWs comprise many heterogeneous subgroups [61]. Therefore, surveillance and interventions need to be adapted to the specific situation and context [44], [61]. Confidentiality and consent are necessary in all stages, as FSWs are a particularly vulnerable population in many countries. A participatory approach that involves these women in surveillance and prevention should be taken [44]. This will probably increase the success of prevention programmes and, furthermore, will empower an often-neglected but important occupational group.

## Supporting Information

Table S1 **Overlap between IDU and sex work, and HIV prevalence.** Summary of studies reporting the proportion of IDUs engaging in sex, FSW injecting drugs and compared HIV prevalence in FSWs according to drug use.
(DOC)

Table S2 **Country data on HIV prevalence and population size.** HIV prevalence in IDU, FSW and the general female population, and the size of the FSW and IDU populations.
(DOC)

Text S1 **Input data retrieved for developing estimates.** Brief description of the range of input data required for estimating the burden of HIV attributable to sex work.
(DOC)

Text S2 **Detailed methods used for estimating the population attributable fraction.** Details on formulas and methods used for estimating population attributable fractions, and equivalence between commonly used formulas.
(DOC)

Text S3 **Detailed methods for estimating attributable fractions of HIV due to female sex work.** Detailed methods and formulas used for estimating the attributable fractions of HIV due to female sex work by two approaches which are used according to data availability.
(DOC)

Text S4 **Multilevel model used for estimating HIV prevalence for the year 2011.** Details on method and formula used for the multilevel modeling of HIV prevalence for the year 2011.
(DOC)
