# Supplementary material for: HIV Due to Female Sex Work: Regional and Global Estimates
Source: PLoS One. 2013 May 23;8(5):e63476. doi: 10.1371/journal.pone.0063476 (PMC3662690; doi:10.1371/journal.pone.0063476)
Supplement: Table S1 — Overlap between IDU and sex work, and HIV prevalence. Summary of studies reporting the proportion of IDUs engaging in sex, FSW injecting drugs and compared HIV prevalence in FSWs according to drug use. (DOC) [file pone.0063476.s001.doc]

# Table S1: Overlap between IDU and sex work, and HIV prevalence

| Region | IDUs engaging in sex work | FSW injecting drugs | HIV prevalence in drug-using and non-drug using FSWs | Reference/ date |
| --- | --- | --- | --- | --- |
| Global | 20-63% of female IDUs (mid-point: 41.5% |  |  | [1], 2008 |
| *Americas* |  |  |  |  |
| Argentina, Brazil | >33% |  |  | [2], 2002 |
| Canada | 4%, 29% |  |  | [3,4], 2002 |
| Mexico |  | 18% ever practiced IDU |  | [5], 2008 |
| *Europe* |  |  |  |  |
| European Multicenter study (Paris, Madrid, Rome, London, Berlin) | 47% of female IDUs |  |  | [6], 2000 |
| Czech Republic |  | 10% |  | [7], 2006 |
| Netherlands |  |  | 1.5% in non-IDU FSWs | [8], 2006 |
| Spain |  |  | 0.6% in non-IDU FSWs | [9], 2004 |
| Belarus, Ukraine |  | 25-30% |  | [10], 2001 |
| Russian Federation |  | <30% |  | [11], 2004 |
| Uzbekistan |  |  | 5% un non-IDU FSWs |  |
| *North Africa, Middle East* |  |  |  |  |
| Lebanon | 12% of female IDUs |  |  | [12], 2010 |
| Syria | 53% |  |  | [13], 2008 |
| Asia |  |  |  |  |
| Bangladesh | 63% of female IDUs |  |  | [14], 2006 |
| China, Sichuan | 60% of female IDUs |  |  | [15], 2006 |
| China, Sichuan |  | 2.5-5% |  | [16], 2005 |
| China, Guangdong | 61% of female IDUs |  |  | [17], 2005 |
| China, Yunnan |  |  | 35.5% in DU FSWs  1.9% in non-DU FSWs | [18], 2010 |
| China, Yunnan | 21% of female IDUs |  |  | [19], 2004 |
| China | 11%* |  |  | [20], 2005 |
| Philippines | 14% |  |  | [21], 2005 |
| Thailand | 7% of female IDUs |  |  | [22], 2005 |
| Viet Nam |  | 20-40% |  | [23], 2005 |
| *Australasia* |  |  |  |  |
| Australia | 35% of female IDUs |  |  | [24], 2005 |

IDU: intravenous drug-using

*: engage in high-risk sexual activities

# References to Table S1

1. Lau JTF, Tsui HY, Zhang Y, Cheng F, Zhang L, et al. (2008) Comparing HIV-related syringe-sharing behaviors among female IDU engaging versus not engaging in commercial sex. Drug Alcohol Depend 97: 54–63. doi:10.1016/j.drugalcdep.2008.03.024.

2. UNAIDS (2002) Sex work and HIV/AIDS. UNAIDS Technical update. Geneva: UNAIDS. Available: http://data.unaids.org/publications/IRC-pub02/jc705-sexwork-tu_en.pdf.

3. Tyndall MW, Patrick D, Spittal P, Li K, O’Shaughnessy MV, et al. (2002) Risky sexual behaviours among injection drugs users with high HIV prevalence: implications for STD control. Sex Transm Infect 78 Suppl 1: i170–175.

4. Gouvernement du Canada, Agence de la santé publique du Canada (2011) Actualités en épidémiologie du VIH/sida - Juillet 2010. Available: http://www.phac-aspc.gc.ca/aids-sida/publication/epi/2010/10-fra.php. Accessed 5 September 2012.

5. Strathdee SA, Philbin MM, Semple SJ, Pu M, Orozovich P, et al. (2008) Correlates of injection drug use among female sex workers in two Mexico-U.S. border cities. Drug Alcohol Depend 92: 132–140. doi:10.1016/j.drugalcdep.2007.07.001.

6. Estebanez PE, Russell NK, Aguilar MD, Beland F, Zunzunegui MV, et al. (2000) Women, drugs and HIV/AIDS: results of a multicentre European study. Int J Epidemiol 29: 734–743. doi:10.1093/ije/29.4.734.

7. Bruckova M, Bautista CT, Graham RR, Maly M, Vandasova J, et al. (2006) HIV infection among commercial sex workers and injecting drug users in the Czech Republic. Am J Trop Med Hyg 75: 1017–1020.

8. EuroHIV (2006) HIV/AIDS Surveillance in Europe. Mid-Year report 2006, No. 74. Paris: French Institute for Public Health Surveillance.

9. European Centre for Disease Prevention and Control (ECDC) - Health Comunication Unit - Eurosurveillance editorial team (2004) Sentinel surveillance of HIV infection in HIV test clinics, Spain 1992-2002. Available: http://www.eurosurveillance.org/ViewArticle.aspx?ArticleId=466. Accessed 21 June 2011.

10. International Harm reduction Program (IHRD), Open Society Institute (2001) Drugs, AIDS and harm reduction. New York: IHRD.

11. Rhodes T, Sarang A, Bobrik A, Bobkov E, Platt L (2004) HIV transmission and HIV prevention associated with injecting drug use in the Russian Federation. Int J Drug Policy 15: 1–16. doi:16/j.drugpo.2003.09.001.

12. Mahfoud Z, Afifi R, Ramia S, Khoury DE, Kassak K, et al. (2010) HIV/AIDS among female sex workers, injecting drug users and men who have sex with men in Lebanon: results of the first biobehavioral surveys. AIDS 24: S45–S54. doi:10.1097/01.aids.0000386733.02425.98.

13. UNAIDS (2008) 2008 Report on the global AIDS epidemic. Geneva: UNAIDS. Available: http://www.unaids.org/en/KnowledgeCentre/HIVData/GlobalReport/2008/2008_Global_report.asp. Accessed 19 August 2010.

14. Azim T, Chowdhury E, Reza M, Ahmed M, Uddin M, et al. (2006) Vulnerability to HIV infection among sex worker and non-sex worker female injecting drug users in Dhaka, Bangladesh: evidence from the baseline survey of a cohort study. Harm Reduct J 3: 33. doi:10.1186/1477-7517-3-33.

15. Choi SYP, Cheung YW, Chen K (2006) Gender and HIV risk behavior among intravenous drug users in Sichuan Province, China. Soc Sci Med 62: 1672–1684. doi:10.1016/j.socscimed.2005.08.046.

16. Monitoring the AIDS epidemic (MAP) (2005) Sex work and HIV/AIDS in Asia. Available: http://www.mapnetwork.org/docs/MAP_SW in Asia Final 04July200405_en.pdf.

17. Lau JTF, Feng T, Lin X, Wang Q, Tsui HY (2005) Needle sharing and sex-related risk behaviours among drug users in Shenzhen, a city in Guangdong, southern China. AIDS Care 17: 166–181. doi:10.1080/09540120512331325662.

18. Jia M, Luo H, Ma Y, Wang N, Smith K, et al. (2010) The HIV Epidemic in Yunnan Province, China, 1989-2007. J Acquir Immune Defic Syndr 53: S34–S40. doi:10.1097/QAI.0b013e3181c7d6ff.

19. UNAIDS, WHO (2004) AIDS epidemic update December 2004. Geneva: UNAIDS.

20. Ministry of Health, People’s Republic of China, UNAIDS, WHO (2006) 2005 Update on the HIV/AIDS epidemic and response in China. Beijing: National Center for AIDS/STD Prevention and Control.

21. National Epidemiology Center, Department of Health (2005) 2005 Integrated HIV Behavioral and Serologic Surveillance. Manila: National Epidemiology Center.

22. Srirak N, Kawichai S, Vongchak T, Razak MH, Jittiwuttikarn J, et al. (2005) HIV infection among female drug users in Northern Thailand. Drug and Alcohol Dependence 78: 141–145. doi:16/j.drugalcdep.2004.10.007.

23. Tran TN, Detels R, Long HT, Lan HP (2005) Drug use among female sex workers in Hanoi, Vietnam. Addiction 100: 619–625. doi:10.1111/j.1360-0443.2005.01055.x.

24. Breen C, Roxburgh A, Degenhardt L (2005) Gender differences among regular injecting drug users in Sydney, Australia, 1996 – 2003. Drug Alcohol Rev 24: 353. doi:10.1080/09595230500263871.
