# Supplementary material for: HIV Due to Female Sex Work: Regional and Global Estimates
Source: PLoS One. 2013 May 23;8(5):e63476. doi: 10.1371/journal.pone.0063476 (PMC3662690; doi:10.1371/journal.pone.0063476)
Supplement: Table S2 — Country data on HIV prevalence and population size. HIV prevalence in IDU, FSW and the general female population, and the size of the FSW and IDU populations. (DOC) [file pone.0063476.s002.doc]

# Table S2: Country data on HIV prevalence and population size

| Country/ region | HIV prevalence in IDU, capital city | HIV prevalence in IDU, capital city | HIV prevalence in FSW | HIV prevalence in FSW | Number of HIV cases in women, 15+ years | Proportion of FSW population | IDU population |
| --- | --- | --- | --- | --- | --- | --- | --- |
|  | Data year | % HIV | Data year | % HIV | - | % of women 15+ years | - |
| Sub-Saharan Africa |  |  |  |  |  |  |  |
| Angola |  |  | 2007 | 23.1 | 110,000 |  |  |
| Benin | 2009 | 4.2 | 2009 | 24.7 | 32,000 |  |  |
| Botswana |  |  |  |  | 170,000 |  |  |
| Burkina Faso |  |  | 2005 | 16.3 | 56,000 |  |  |
| Burundi |  |  | 2007 | 39.8 | 90,000 |  |  |
| Cameroon |  |  | 2009 | 35.5 | 320,000 |  |  |
| Central African Republic |  |  |  |  | 67,000 |  |  |
| Chad |  |  | 2009 | 20.0 | 110,000 |  |  |
| Comoros |  |  |  |  | <100 |  |  |
| Congo |  |  | 2007 | 0.0 | 40,000 |  |  |
| Côte d'Ivoire |  |  | 2007 | 14.7 | 220,000 | 2.9 |  |
| Democratic Republic of the Congo |  |  |  |  |  |  |  |
| Djibouti |  |  | 2008 | 20.3 | 7,400 |  |  |
| Equatorial Guinea |  |  |  |  | 11,000 |  |  |
| Eritrea |  |  | 2008 | 7.8 | 13,000 |  |  |
| Ethiopia |  |  |  |  | NA |  |  |
| Gabon |  |  | 2010 | 23.6 | 25,000 |  |  |
| Gambia |  |  |  |  | 9,700 |  |  |
| Ghana |  |  | 2009 | 25.0 | 140,000 |  | 1,000 |
| Guinea |  |  | 2008 | 32.7 | 41,000 | 3.6 |  |
| Guinea-Bissau |  |  | 2009 | 39.6 | 12,000 |  |  |
| Kenya | 2004 | 42.9 |  |  | 760,000 | 5.5 | 130,748 |
| Lesotho |  |  |  |  | 160,000 |  |  |
| Liberia |  |  |  |  | 19,000 |  |  |
| Madagascar |  |  | 2007 | 0.5 | 7,300 | 3.6 |  |
| Malawi |  |  | 2006 | 70.7 | 470,000 |  |  |
| Mali |  |  | 2006 | 35.3 | 40,000 |  |  |
| Mauritania |  |  | 2010 | 32.6 | 4,000 |  |  |
| Mauritius | 2009 | 47.1 |  |  | 2,500 |  | 17,500 |
| Mozambique |  |  |  |  | 760,000 |  |  |
| Namibia |  |  |  |  | 95,000 |  |  |
| Niger |  |  | 2009 | 35.6 | 28,000 | 1.4 | 1,000 |
| Nigeria | 2010 | 4.2 | 2007 | 32.7 | 1,700,000 | 8.7 | 5,000 |
| Rwanda |  |  | 2010 | 24.0 | 88,000 |  |  |
| Senegal | 2011 | 9.2 | 2006 | 19.8 | 32,000 |  |  |
| Sierra Leone |  |  | 2005 | 8.5 | 28,000 | 5.3 |  |
| Somalia |  |  | 2008 | 5.5 | 15,000 |  | 1,000 |
| South Africa | 2004 | 12.4 |  |  | 3,300,000 |  | 262,975 |
| Swaziland |  |  |  |  | 100,000 |  |  |
| Togo |  |  | 2005 | 44.5 | 67,000 |  |  |
| Uganda |  |  | 2010 | 37.2 | 610,000 |  |  |
| United Republic of Tanzania |  |  |  |  | 730,000 |  |  |
| Zambia |  |  | 2004 | 65.4 | 490,000 |  |  |
| Zimbabwe |  |  |  |  | 620,000 |  |  |
| Cape Verde |  |  |  |  |  |  |  |
| Sao Tome and Principe |  |  |  |  |  |  |  |
| Seychelles |  |  |  |  |  |  |  |
| Latin America |  |  |  |  |  |  |  |
| Argentina | 2007 | 6.7 | 2008 | 1.9 | 36000 |  | 65,829 |
| Belize |  |  |  |  | 2600 | 5.9 |  |
| Bolivia |  |  | 2007 | 0.4 | 3600 | 0.2 | 200 |
| Brazil | 2000 | 46 |  |  | 220000 |  | 800,000 |
| Chile |  |  | 2006 | 0.9 | 12000 |  | 42,176 |
| Colombia | 1999 | 1.4 | 2008 | 1.6 | 50000 | 0.5 | 5,000 |
| Costa Rica |  |  | 2005 | 0.1 | 2800 |  | 1,000 |
| Ecuador |  |  |  |  | 11000 |  | 9,270 |
| El Salvador |  |  | 2009 | 4.1 | 11000 |  | 4,437 |
| Guatemala |  |  | 2006 | 1.0 | 20000 |  | 7,363 |
| Guyana |  |  | 2009 | 16.6 | 2800 |  |  |
| Honduras |  |  | 2006 | 2.3 | 12000 |  | 4,335 |
| Mexico | 2005 | 4.1 | 2009 | 0.9 | 59000 |  | 53,162 |
| Nicaragua | 2000 | 6 | 2006 | 2.2 | 2100 |  | 3,424 |
| Panama |  |  | 2001 | 1.8 | 6300 |  | 2,090 |
| Paraguay | 2006 | 9.1 | 2008 | 1.8 | 3800 |  | 3,825 |
| Peru |  |  | 2006 | 0.5 | 18000 | 0.2 | 500 |
| Suriname |  |  | 2006 | 24.1 | 1100 |  |  |
| Uruguay |  |  |  |  | 3100 |  | 2,192 |
| Venezuela (Bolivarian Republic of) |  |  |  |  |  | 1.1 | 1,500 |
| North America |  |  |  |  |  |  |  |
| Canada | 2008 | 12.7 |  |  | 14000 |  | 286,987 |
| United States of America |  |  |  |  | 310000 |  | 1,857,354 |
| Asia, East |  |  |  |  |  |  |  |
| China | 2009 | 9.3 | 2009 | 0.6 | 230000 | 0.64 | 2,350,000 |
| Democratic People's Republic of Korea |  |  |  |  |  |  |  |
| Japan |  |  |  |  | 2700 |  |  |
| Mongolia |  |  | 2011 | 0.0 | <200 |  | 63 |
| Republic of Korea |  |  |  |  | 2900 |  | 3,000 |
| Asia, South/South-East |  |  |  |  |  |  |  |
| Afghanistan | 2005 | 4.9 | 2009 | 0.2 | <100 |  | 6,900 |
| Bangladesh | 2011 | 1.10 | 2011 | 0.3 | 1900 |  | 30,000 |
| Bhutan |  |  |  |  | <500 |  |  |
| Brunei Darussalam |  |  |  |  | <100 |  | 3,155 |
| Cambodia | 2011 | 24.1 | 2011 | 13.9 | 35000 | 0.2 | 1,750 |
| India | 2009 | 9.2 | 2009 | 4.9 | 880000 | 0.3 | 164,820 |
| Indonesia | 2011 | 36.4 | 2011 | 9.0 | 88000 | 0.3 | 219,130 |
| Iran (Islamic Republic of) | 2005 | 20.5 |  |  | 26000 |  | 180,000 |
| Lao People's Democratic Republic |  |  | 2011 | 1.0 | 3500 |  | 1,700 |
| Malaysia | 2009 | 22.1 | 2009 | 10.7 | 11000 | 0.004 | 205,000 |
| Maldives |  |  |  |  | <100 | 0.01 | 410 |
| Myanmar | 2011 | 21.91 | 2011 | 9.4 | 81000 |  | 75,000 |
| Nepal | 2012 | 6.3 | 2012 | 1.7 | 20000 | 1.6 | 22,050 |
| Pakistan | 2011 | 27.2 | 2011 | 0.6 | 28000 |  | 130,460 |
| Philippines | 2011 | 13.6 | 2011 | 0.3 | 2600 | 2.0 | 17,000 |
| Singapore |  |  | 2011 | 0.0 | 1000 |  | 15,000 |
| Sri Lanka | 2011 | 0 | 2011 | 0.2 | <1000 |  | 27,827 |
| Thailand | 2010 | 21.9 | 2011 | 1.8 | 210000 |  | 160,528 |
| Timor-Leste |  |  | 2010 | 2.8 | NA |  | 105 |
| Viet Nam | 2011 | 13.4 | 2011 | 3.0 | 81000 | 0.1 | 135,305 |
| Caribbean |  |  |  |  |  |  |  |
| Bahamas |  |  |  |  | 3700 |  |  |
| Barbados |  |  |  |  | <1000 |  |  |
| Cuba |  |  | 2009 | 0.1 | 2200 |  | 8,255 |
| Dominican Republic |  |  | 2008 | 4.8 | 32000 | 1.3 | 110 |
| Haiti |  |  | 2009 | 5.3 | 67000 | 1.6 |  |
| Jamaica |  |  | 2009 | 4.9 | 10000 |  |  |
| Trinidad and Tobago |  |  |  |  | 4700 |  |  |
| Antigua and Barbuda |  |  |  |  |  |  |  |
| Dominica |  |  |  |  |  |  |  |
| Grenada |  |  |  |  |  |  |  |
| Saint Kitts and Nevis |  |  |  |  |  |  |  |
| Saint Lucia |  |  |  |  |  |  |  |
| Saint Vincent and the Grenadines |  |  |  |  |  |  |  |
| Europe, East/Asia, Central |  |  |  |  |  |  |  |
| Armenia | 2005 | 9.3 | 2005 | 0.4 | <1000 | 0.6 | 2,000 |
| Azerbaijan | 2008 | 10.3 | 2008 | 1.7 | 2100 | 1.1 | 300,000 |
| Belarus | 2009 | 13.7 | 2009 | 6.4 | 8300 | 0.3 | 6,308 |
| Bosnia and Herzegovina |  |  |  |  |  | 0.3 | 11,458 |
| Bulgaria | 2008 | 6.8 | 2008 | 0.7 | 1100 | 0.3 | 20,250 |
| Croatia | 2006 | 0.6 | 2006 | 1.4 | <500 | 0.2 | 20,942 |
| Estonia | 2007 | 62.5 | 2006 | 7.7 | 3000 | 0.5 | 13,801 |
| Georgia | 2008 | 2.2 | 2009 | 1.3 | 1500 | 0.4 | 127,833 |
| Kazakhstan | 2009 | 2.9 | 2009 | 1.3 | 7700 | 0.5 | 100,000 |
| Kyrgyzstan | 2009 | 14.3 | 2009 | 1.6 | 2800 | 0.2 | 25,000 |
| Latvia | 2007 | 22.6 | 2001 | 7.7 | 2600 | 0.7 | 18,725 |
| Lithuania | 2008 | 8.0 | 2000 | 1.4 | <500 | 0.4 | 5,123 |
| Republic of Moldova | 2001 | 17.0 | 2005 | 8.5 | 5100 |  | 3,810 |
| Romania | 2006 | 1.4 | 2005 | 1.4 | 4700 | 0.4 | 100,970 |
| Russian Federation | 2009 | 15.6 | 2009 | 4.5 | 480000 | 0.3 | ,825,000 |
| Tajikistan | 2008 | 17.6 | 2008 | 2.8 | 2700 | 0.2 | 17,000 |
| Turkmenistan |  |  |  |  |  | 0.1 | 11,148 |
| Ukraine | 2009 | 22.9 | 2005 | 8.0 | 170000 | 0.2 | 375,000 |
| Uzbekistan | 2009 | 18.4 | 2009 | 3.2 | 8000 | 0.2 | 80,000 |
| Europe, West/Central |  |  |  |  |  |  |  |
| Albania |  |  | 2010 | 1.1 |  | 0.5 | 19,500 |
| Austria | 2009 | 4.0 |  |  | 4600 | 0.5 | 17,500 |
| Belgium | 2008 | 8.7 | 2009 | 0.4 | 4400 | 0.2 | 25,400 |
| Czech Republic | 2009 | 0.1 | 2005 | 0.7 | <1000 | 0.2 | 29,000 |
| Denmark | 2006 | 2.1 |  |  | 1400 | 0.2 | 15,416 |
| Finland | 2009 | 0.7 |  |  | <1000 | 0.1 | 15,650 |
| France | 2006 | 12.2 |  |  | 48000 | 0.1 | 122,000 |
| Germany | 2006 | 2.9 |  |  | 12000 | 0.7 | 94,250 |
| Greece | 2006 | 0.5 |  |  | 2700 | 0.2 | 9,720 |
| Hungary | 2000 | 2.2 |  |  | <1000 | 0.3 | 3,941 |
| Iceland |  |  |  |  | <200 |  |  |
| Ireland | 1999 | 5.8 |  |  | 2000 |  | 6,289 |
| Italy | 2006 | 12.1 |  | 0.0 | 48000 | 0.2 | 326,000 |
| Luxembourg | 2008 | 1.8 |  |  | <500 | 0.2 | 1,715 |
| Malta | 2006 | 0.0 |  |  |  |  |  |
| Montenegro |  |  |  |  |  |  |  |
| Netherlands | 2002 | 9.5 | 2005 | 6.5 | 6900 | 0.3 | 3,115 |
| Norway | 2005 | 0.4 |  |  | 1200 | 0.2 | 10,049 |
| Poland | 2006 | 8.9 | 2002 | 2 | 8200 | 0.3 | 96,514 |
| Portugal | 2008 | 14.0 |  |  | 13000 |  | 32,287 |
| Serbia and Montenegro | 2008 | 4.8 |  |  | 2000 | 0.3 | 30,000 |
| Slovakia | 2006 | 0.0 |  |  | <100 | 0.2 | 18,841 |
| Slovenia | 2004 | 0.4 |  |  | <200 | 0.7 | 7,310 |
| Spain | 2008 | 19.5 | 2008 | 0.9 | 32000 | 0.3 | 83,972 |
| Sweden | 2007 | 5.4 |  |  | 2500 | 0.0 |  |
| Switzerland | 2006 | 10.9 |  |  | 5700 |  | 31,653 |
| The former Yugoslav Republic of Macedonia | 2006 | 0.8 | 2006 | 0 |  | 0.4 |  |
| United Kingdom | 2006 | 2.3 |  |  | 26000 | 0.3 | 156,398 |
| Andorra |  |  |  |  |  |  |  |
| Monaco |  |  |  |  |  |  |  |
| San Marino |  |  |  |  |  |  |  |
| North Africa/Middle East |  |  |  |  |  |  |  |
| Algeria |  |  |  |  | 5200 |  | 40,961 |
| Bahrain | 2000 | 0.3 |  |  |  |  | 674 |
| Cyprus | 2006 | 0.0 |  |  |  |  | 305 |
| Egypt | 2006 | 3.5 | 2006 | 0.9 | 2400 |  | 88,618 |
| Iraq |  |  |  |  |  |  | 34,673 |
| Israel | 2005 | 4.0 |  |  | 2200 |  |  |
| Jordan |  |  |  |  |  |  | 4,850 |
| Kuwait |  |  |  |  |  |  | 4,100 |
| Lebanon |  |  | 2008 | 0.0 | 1100 |  | 3,300 |
| Libyan Arab Jamahiriya | 2004 | 22.0 |  |  |  |  | 1,685 |
| Morocco | 2009 | 2.1 | 2009 | 2.4 | 8100 |  | 18,500 |
| Oman | 2000-2005 | 11.8 |  |  | <500 |  | 4,250 |
| Qatar |  |  |  |  | <100 |  | 1,190 |
| Saudi Arabia |  |  |  |  |  |  | 23,600 |
| Sudan | 2003 | 0.0 | 2008 | 5.5 | 140000 |  | 37,828 |
| Syrian Arab Republic |  |  |  |  |  |  | 6,000 |
| Tunisia | 2009 | 3.1 | 2009 | 0.4 | 1000 |  | 13,163 |
| Turkey |  |  |  |  | 1400 |  | 99,887 |
| United Arab Emirates |  |  |  |  |  |  | 4,800 |
| Yemen |  |  | 2011 | 0.0 |  |  | 19,770 |
| Oceania |  |  |  |  |  |  |  |
| Australia | 2008 | 1.5 | 2008 | 0.1 | 6200 | 0.001 | 149,591 |
| Fiji |  |  |  |  | <200 |  | 131 |
| New Zealand | 2006 | 1.6 | 2007 | 0.0 | <1000 |  | 20,163 |
| Papua New Guinea |  |  | 2009 | 7.4 | 18000 | 0.01 | 7,500 |
| Cook Islands |  |  |  |  |  |  |  |
| Kiribati |  |  |  |  |  |  |  |
| Marshall Islands |  |  |  |  |  |  |  |
| Micronesia (Federated States of) |  |  |  |  |  |  |  |
| Nauru |  |  |  |  |  |  |  |
| Niue |  |  |  |  |  |  |  |
| Palau |  |  |  |  |  |  |  |
| Samoa | 2004-2005 | 0.0 |  |  |  |  |  |
| Solomon Islands | 2004-2005 | 0.0 |  |  |  |  |  |
| Tonga | 2004-2005 | 0.0 |  |  |  |  |  |
| Tuvalu |  |  |  |  |  |  |  |
| Vanuatu |  |  |  |  |  |  |  |

**Sources of values in Table A2**

- International Harm reduction Program (IHRD), Open Society Institute. Harm reduction developments 2008. Countries with injection-driven HIV epidemics. New York: IHRD; 2008.
- Aceijas C, Friedman SR, Cooper HLF, et al. Estimates of injecting drug users at the national and local level in developing and transitional countries, and gender and age distribution. Sex Transm Infect. 2006;82 Suppl 3:iii10-17.
- Vandepitte J, Lyerla R, Dallabetta G, et al. Estimates of the number of female sex workers in different regions of the world. Sex Transm Infect. 2006;82 Suppl 3:iii18-25.
- National Epidemiology Center, Department of Health. *2005 Integrated HIV Behavioral and Serologic Surveillance*. Manila: National Epidemiology Center; 2005.
- Breen C, Roxburgh A, Degenhardt L. Gender differences among regular injecting drug users in Sydney, Australia, 1996 – 2003. Drug and Alcohol Review. 2005;24(4):353.
- MoH, Report of a Consensus workshop, HIV estimates and projections for Cambodia, 2006-2010, Surveillance Unit, Phnom Penh, 2007.
- Mathers BM, Degenhardt L, Phillips B, et al. Global epidemiology of injecting drug use and HIV among people who inject drugs: a systematic review. Lancet. 2008;372(9651):1733-1745.
- Canada’s UNGASS 2010 Report, Government of Canada, Report to the Secretary General of the United Nations on the United Nations General Assembly Special Session on HIV/AIDS, Declaration of Commitment on HIV/AIDS, January 2008 – December 2009.
- McInnes C, Druyts E, Harvard S, et al. HIV/AIDS in Vancouver, British Columbia: A growing epidemic. *Harm Reduction Journal*. 2009;6(5). Available at: http://www.harmreductionjournal.com/content/6/1/5. Accessed May 9, 2011.
- Bruckova M, Bautista CT, Graham RR, et al. Short report: HIV infection among commercial sex workers and injecting drug users in the Czech Republic. *Am. J. Trop. Med. Hyg.* 2006;75(5):1017–1020.
- EuroHIV. *HIV/AIDS Surveillance in Europe. Mid-Year report 2006, No. 74*. Paris: French Institute for Public Health Surveillance. 2006.
- Baral S, Beyrer C, Muessig K, et al. Burden of HIV among female sex workers in low-income and middle-income countries: a systematic review and meta-analysis. *Lancet Infect Dis*. 2012;12(7):538–549.
- Malta M, Magnanini MM, Mello MB, et al. HIV prevalence among female sex workers, drug users and men who have sex with men in Brazil: A Systematic Review and Meta-analysis. *BMC Public Health*. 2010;10(1):317.
- UNAIDS. *2012 UNAIDS Report on the Global Aids epidemic*. Geneva: UNAIDS; 2012.
- UNAIDS. *2010 Report on the global AIDS epidemic*. Geneva: UNAIDS; 2010.
- UNAIDS. *2008 Report on the global AIDS epidemic*. Geneva: UNAIDS; 2008.
- UNGASS country reports available on: http://www.unaids.org/en/regionscountries/countries/
