# Supplementary material for: HIV Due to Female Sex Work: Regional and Global Estimates
Source: PLoS One. 2013 May 23;8(5):e63476. doi: 10.1371/journal.pone.0063476 (PMC3662690; doi:10.1371/journal.pone.0063476)
Supplement: Text S1 — Input data retrieved for developing estimates. Brief description of the range of input data required for estimating the burden of HIV attributable to sex work. (DOC) [file pone.0063476.s003.doc]

# Text S1: Input data retrieved for developing estimates

The proportion of HIV in the general female adult population ranged from <0.01 to 26% (median 0.2%). Countries with the highest HIV prevalence in their general adult female population were Swaziland, Lesotho and Botswana. HIV prevalence in high-risk groups ranged from <0.01 to 74% in FSWs and from <0.01 to 63% in PWID (median 4 and 6% respectively). The highest proportions of HIV infected FSWs were found in Sub Saharan Africa, especially in Botswana, Lesotho and Swaziland. The percentage of FSWs in the general adult female population ranged from <0.01 to 9% (median 0.3). Between 0.5 and 55% of PWID were female (median 16%). The proportion of PWID among the general female adult population ranged from <0.01 to 0.5% (median 0.03%).
