# Supplementary material for: HIV Due to Female Sex Work: Regional and Global Estimates
Source: PLoS One. 2013 May 23;8(5):e63476. doi: 10.1371/journal.pone.0063476 (PMC3662690; doi:10.1371/journal.pone.0063476)
Supplement: Text S2 — Detailed methods used for estimating the population attributable fraction. Details on formulas and methods used for estimating population attributable fractions, and equivalence between commonly used formulas. (DOC) [file pone.0063476.s004.doc]

# Text S2: Detailed methods used for estimating the population attributable fraction

The formula we used for the PAF to estimate the fraction of cases (exposed and unexposed to female sex work) that could have been avoided if exposure had not occurred is the following:

$PAF_{FSW}=\frac{\text{(PR }_{\text{FSW}}\text{ - PR }_{\text{unexp}}\text{) × prop }_{\text{FSW}}\text{× n }_{\text{female}}}{N_{HIV}}$ (1)

where *PR _FSW_* is the prevalence of HIV in FSWs, *PR _unexp_* the prevalence of HIV in the female population not engaging in sex work, *prop _FSW_* the proportion of FSWs in the general female adult population, *n _female_* the total female adult population, and *N_HIV_* the total number of HIV-positive women. This formula can also be interpreted as the excess cases of HIV in FSW divided by the total HIV cases in the adult female population.

In formula (1), disease prevalence used as a measure of risk to describe the relative importance of the exposure on the current disease burden is useful for a chronic condition like HIV. Formula (1) can in this case be written as follows, as used in the literature [1,2]:

$PAF=\frac{(R_{\exp}-R_{un\exp})\times n_{\exp}}{n_{t}\times R_{t}}$ (2)

where *R _exp_* is the risk in the exposed, *R _unexp_* the risk in the unexposed, *n _exp_* is the number of people exposed, *n _t_* the total number of people in the population, and *R _t_* the risk in the total population. Furthermore, we have the following relations:

$RR=\frac{R_{\exp}}{R_{un\exp}}$ (3) and

$\text{R}_{\text{ t}}\text{=(1-p}_{\text{ exp}}\text{)×R}_{\text{ unexp}}\text{+p}_{\text{ exp}}\text{ × R}_{\text{t}}$ (4)

where *RR* is the risk ratio, and *p _exp_* the proportion of the population exposed. When using equations (3) and (4) in equation (2), the frequently used equation (5) is obtained [1,2].

$PAF=\frac{p_{\exp}(RR-1)}{p_{\exp}(RR-1)+1}$ (5)

By similar replacements, the following expression which is also frequently used can be obtained [2]:

$PAF=\frac{R_{t}-R_{un\exp}}{R_{t}}$ (6)

# References to Text S2

1. Rothman KJ, Greenland S (1998) Modern Epidemiology. 2nd ed. Lippincott Williams & Wilkins. 737 p.

2. Last JM, editor (2000) A Dictionary of Epidemiology. 4th ed. Oxford University Press, USA. 224 p.
