# Supplementary material for: HIV Due to Female Sex Work: Regional and Global Estimates
Source: PLoS One. 2013 May 23;8(5):e63476. doi: 10.1371/journal.pone.0063476 (PMC3662690; doi:10.1371/journal.pone.0063476)
Supplement: Text S3 — Detailed methods for estimating attributable fractions of HIV due to female sex work. Detailed methods and formulas used for estimating the attributable fractions of HIV due to female sex work by two approaches which are used according to data availability. (DOC) [file pone.0063476.s005.doc]

# Text S3: Detailed methods for estimating attributable fractions of HIV due to female sex work

We calculated the PAFs using two approaches, depending on data availability for countries:

*Method 1*

Method 1 was used for all countries not located in Western and Central Europe because for these countries we did not have information on HIV in FSWs practicing IDU. This method involved three steps.

First, we calculated the country PAF of HIV prevalence due to exposure to female sex work (PAF _FSW_) without adjusting for risk due to IDU (equation 1):

$PAF_{FSW}=\frac{\text{(PR}_{\text{FSW}}\text{ - PR}_{\text{unexp}}\text{ ) × prop }_{\text{FSW}}\text{× n}_{\text{female}}}{N_{HIV}}$ (1)

*PR _FSW_* is the prevalence of HIV in FSWs, *PR _unexp_* is the prevalence of HIV in the female population not engaging in sex work, *prop _FSW_* is the proportion of FSWs in the general female adult population, *n _female_* is the total female adult population and *N _HIV_* is the total number of HIV-positive women. The PAF here corresponds to the excess cases in FSW divided by the total HIV cases in the adult female population. Correspondence with other commonly used formulas for the PAF is provided in the Supplementary Webappendix.

Secondly, we estimate the country PAF of HIV infections in the general female adult population that is due to intravenous drug use in FSWs (*PAF FSW_injectdrugs*, equation 2):

(2)

*PR _PWID_* is the prevalence of HIV in PWID, *PR _unexp_* is the prevalence of HIV in the unexposed, approximated here by the HIV prevalence in the general population, *n _PWID_* is the total number of people who inject drugs, *prop PWID_female* is the female proportion of PWIDs and *prop PWID_sexwork* is the proportion of PWID who declared having sold sex in the last year.

In the third step we deduct the PAF of HIV prevalence due to drug-injection and female sex work (approximated by estimated HIV cases in PWID who reported having sold sex in the last year) from the PAF of HIV prevalence due to (unadjusted) female sex work (equation 3). We thereby estimate the PAF of HIV prevalence in the general female adult population that is attributable to female sex work having accounted for injecting drug use in FSWs (*PAF FSW_adjusted*).

$\text{PAF }_{\text{FSW\_adjusted}}\text{=PAF }_{\text{FSW}}\text{- PAF }_{\text{FSW\_injectdrugs}}$ (3)

*Method 2*

HIV prevalence data in non-injecting FSWs (‘*PR _FSWnoIDU_*’) were available for West and Central Europe and were taken from two representative sources for this region [1,2]. For this region (17 countries) option 2 was preferred over option 1, since no assumptions are made about the relationship between sex work, IDU and risk for HIV infection. The adjusted PAF of HIV infections due to female sex work can be directly (equation 4):

$PAF_{FSWadjusted}= \frac{(PR_{FSWnoIDU}-PR_{un\exp})\times prop_{FSWnoIDU}\times n_{female}}{N_{HIV}}$ (4)

*PR _FSWnoIDU_* is the HIV prevalence in FSWs not practicing IDU, and *prop _FSWnoIDU_* the proportion of FSW not injecting drugs in the general female population. The PAF here corresponds to the excess cases in FSW not injecting drugs divided by the total HIV cases in the adult female population.

# References to Text S3

1. Barrasa A, Castilla J, Del Romero J, Pueyo I, De Armas C, et al. (2004) Sentinel surveillance of HIV infection in HIV test clinics, Spain 1992-2002. Available: http://www.eurosurveillance.org/ViewArticle.aspx?ArticleId=466. Accessed 11 September 2012.

2. EuroHIV (2006) HIV/AIDS Surveillance in Europe. Mid-Year report 2006. Paris: French Institute for Public Health Surveillance.
