# Supplementary material for: HIV Due to Female Sex Work: Regional and Global Estimates
Source: PLoS One. 2013 May 23;8(5):e63476. doi: 10.1371/journal.pone.0063476 (PMC3662690; doi:10.1371/journal.pone.0063476)
Supplement: Text S4 — Multilevel model used for estimating HIV prevalence for the year 2011. Details on method and formula used for the multilevel modeling of HIV prevalence for the year 2011. (DOC) [file pone.0063476.s006.doc]

# Text S4: Multilevel model used for estimating HIV prevalence for the year 2011

The two level model to estimate HIV prevalence in FSWs was built as follows:

$Y_{ij}=\beta_{0}+\beta_{1}X_{1ij}+\sum_{k=2}^{15} \beta_{k}X_{kj}+\mu_{j}+\varepsilon_{ij}$ (1)

where *Y_ij_* is the HIV proportion in FSWs for the *i-*th year in the *j-*th country, *β_0_* is the overall mean intercept of *Y* across all groups, *β_1_* is the regression coefficient for the covariate year (*X_1_ij*), *β_k_* are the regression coefficients for the regional covariate (*X_kj_*), *μ_j_* are the residuals on the country level (group-level residuals) and *ε_ij_* are the residuals at the level of the observations (individual-level residuals) [1].

# References to Text S4

1. Hox J (n.d.) Multilevel Analysis, Techniques and Applications. 2nd ed. New York: Routledge.
